# 16S rRNA Gene Copy Number Normalization Does Not Provide More Reliable Conclusions in Metataxonomic Surveys

**DOI:** 10.1007/s00248-020-01586-7

**Published:** 2020-08-29

**Authors:** Robert Starke, Victor Satler Pylro, Daniel Kumazawa Morais

**Affiliations:** 1 Laboratory of Environmental Microbiology, Institute of Microbiology of the Czech Academy of Sciences, Praha, Czech Republic; 2 Department of Biology, Federal University of Lavras—UFLA, Lavras, Minas Gerais Brazil; 3 Bioinformatics Core Facility, Institute of Microbiology of the Czech Academy of Sciences, Praha, Czech Republic

**Keywords:** 16S rRNA, Metataxonomic surveys, Gene

## Abstract

Sequencing 16S rRNA gene amplicons is the gold standard to uncover the composition of prokaryotic communities. The presence of multiple copies of this gene makes the community abundance data distorted and gene copy normalization (GCN) necessary for correction. Even though GCN of 16S data provided a picture closer to the metagenome before, it should also be compared with communities of known composition due to the fact that library preparation is prone to methodological biases. Here, we process 16S rRNA gene amplicon data from eleven simple mock communities with DADA2 and estimate the impact of GCN. In all cases, the mock community composition derived from the 16S sequencing differs from those expected, and GCN fails to improve the classification for most of the analysed communities. Our approach provides empirical evidence that GCN does not improve the 16S target sequencing analyses in real scenarios. We therefore question the use of GCN for metataxonomic surveys until a more comprehensive catalogue of copy numbers becomes available.

**Electronic supplementary material:**

The online version of this article (10.1007/s00248-020-01586-7) contains supplementary material, which is available to authorized users.

Amplicon sequencing of 16S rRNA gene is considered a gold standard to evaluate the composition of prokaryotic communities due to (i) low cost, (ii) easy availability, (iii) easy practicality of extraction and preparation kits, (iv) high taxonomic resolution as deep as the level of genera (or sometimes species) and (v) extensive databases. The concept of gold standards implies a level of perfection never attained by any biological test [1] which is why those are constantly challenged and replaced when appropriate [2]. Still, amplicon sequencing outcompetes (88,889 papers with “16S rRNA” as of June 9, 2020) other possible techniques to describe the community structure such as metagenomics (22,106), metaproteomics (1717) or metatranscriptomics (2639) with many thousand publications in recent years. The general practice as shown by the myriads of publications does not comprise the correction of the obtained raw counts by 16S rRNA gene copy numbers per bacterial genome even though it is known that bacteria can have multiple copy numbers of the 16S rRNA gene and the normalization of 16S rRNA amplicon data gave a picture closer to the metagenomes [3]. However, both amplicon and shotgun sequencing are prone to methodological biases introduced by extraction, PCR, sequencing and bioinformatics and could thus similarly diverge from the real picture [4]. Recently, it was recommended not to use GCN based on the systematic evaluation of the predictability of 16S GCNs in bacteria [5], but the validity of GCN in 16S rRNA gene amplicon sequencing has never been shown for communities with known composition. These so-called mock communities are defined mixtures of microbial cells or nucleic acids created in vitro for the simulation of the composition of a microbiome sample, or DNA mixture isolated therefrom are used as a uniform benchmark for microbiome and metagenome technology development and evaluation [6]. We believe that the comparison of amplicon data to the actual relative abundances of taxonomic groups in the community is the only way to verify the validity of GCN in 16S rRNA gene amplicon data. Admittedly, the choice of the mock community (from cells, DNA, RNA, proteins or metabolites), the sample preparation protocol, the primer pair that targets a specific region of the 16S rRNA gene and the processing pipeline can all bias the outcome and must all be considered moving forward.

Here, we processed nine bacterial mock communities from purified genomic DNA and two from cloned 16S rRNA genes in the pUC19 plasmid vector targeting the V4 region of the 16S rRNA gene provided elsewhere [7]. We used *DADA2* v1.8 [8] for sequence data processing and the amplicon sequence variants (ASV) classification using the naïve Bayesian classifier method and the SILVA database (version 138) as reference [9], with or without GCN correction, based on the information available in the Ribosomal Database Project (*RDP*, Release 11, Update 5 from September 30, 2016) [10]. All applied methods are provided in detail as Supplementary Information.

Many pipelines exist for the processing and taxonomic annotation of 16S rRNA gene amplicon data [11, 12], but all taxonomic assignment methods are similarly limited which is why the use of a taxonomical assignment on a higher rank than species ensures both better accuracy and the detection of species without an exact match [8]. Approaches based on machine learning such as TAGME (https://github.com/gabrielrfernandes/tagme) can provide better assignments as the exact matching used by *DADA2* yields several ambiguities but are as yet unpublished. The community profile derived from ASVs [13] appeared to better the expected profile than those based on a clustering method, as we reported previously in a study using three of these mock communities [14]. The strategy we used, however, harbours issues with varying gene copy numbers within the same genus that forces GCN to use averages. In addition to that, multiple copies in the same gene can diverge [15] as a result of pseudogene formation or horizontal gene transfer [16]. One of the eleven mock communities (Mock-12) showed a poor fit of the sequencing data to the mock community (shown in red in Table 1) and was therefore removed from the analysis (Fig. 1, legend is shown in Supplementary Fig. S1). Interestingly, the richness of the mock communities was overrepresented by, on average, 27.4% (*n* = 10, SE = 12.1%) in the sequencing data, but this was mainly caused by low-abundant genera, making up 1.4% (*n* = 10, SE = 0.1%) of the ASV counts (Fig. 1). Another cause of misidentification are unidentified sequences that made up 4.0% (*n* = 10, SE = 3.1%). However, *DADA2* with *RDP* seemed to annotate the amplicon data more reliably as *Escherichia* was not mistakenly identified as *Klebsiella* within the family *Enterobacteriaceae* when operational taxonomic units (OTUs) were previously annotated using *Blast* [14]. Similar to the approach with blasting OTUs [14], many genera such as *Bacteroides* aligned better with the expected content of the mock community with normalization by GCN but other genera such as *Escherichia* or *Nitrosomonas* aligned better without GCN. Altogether, the 16S sequencing data without GCN fitted the mock community composition 7.1% (*n* = 10, SE = 3.6%) better than with GCN (Fig. 1). This was driven by Mock-18 where *Nitrosomonas* and *Desulfovibrio* were misidentified and by Mock-19 where the unidentified ASVs decreased the fitness GCN as the average copy number of bacteria was applied. Both mock communities derived from cloned 16S rRNA genes in the pUC19 plasmid vector while the mock communities from purified DNA showed smaller RSS to the actual community composition. As expected, unidentified ASVs will be overrepresented in the normalized data, while the bacteria with known gene copy numbers could have more, in this case, an average of 6.6 (*n* = 7, SE = 0.9) in Mock-19. The misrepresentation of the mock community increases with an increasing number of unidentified ASVs, which can further be improved as soon as better classification methods arise.

**Table 1:** Misidentification as unidentified ASVs (NA) and ASVs assigned to other genera, the Shannon diversity and richness of bacterial genera as well as the residual sum of squares (RSS) as discrepancy of the sequenced community composition and the structure of the mock community on genus level

Community	Misidentification		Shannon diversity			Richness		RSS to Mock	
	NA	Other genera	Mock	Raw	GCN	Mock	Raw	Raw	GCN
Mock-12	0.0003	0.0461	2.0736	0.3926	0.5765	11	13	**1.3138**	1.3274
Mock-13	0.0005	0.0057	2.8216	2.6968	2.7120	18	32	**0.5198**	0.5258
Mock-14	0.0003	0.0103	2.8216	2.7039	2.7456	18	35	0.5245	**0.4991**
Mock-15	0.0001	0.0038	2.8216	2.6950	2.6591	18	30	**0.5447**	0.5823
Mock-16	0.0853	0.0913	3.7543	3.1574	3.0887	46	54	**0.8441**	0.9053
Mock-18	0.0019	0.0000	2.7081	2.6027	2.4329	15	15	**0.3089**	0.5965
Mock-19	0.3074	0.0000	2.3581	2.4581	2.1697	15	15	**0.8829**	1.1353
Mock-20	0.0000	0.0001	2.7616	2.5335	2.4519	17	17	**0.5107**	0.5766
Mock-21	0.0000	0.0000	1.6901	1.5246	1.5091	17	14	0.4041	**0.3534**
Mock-22	0.0004	0.0292	2.7616	2.7212	2.7024	17	20	**0.2978**	0.4075
Mock-23	0.0008	0.0017	1.6901	1.7205	1.7214	17	20	0.1938	**0.1566**

The mock community (Mock) was compared with the 16S amplicon sequencing data without (raw) or with gene copy normalization (GCN). Mock-12 was removed from the analysis due to the low Shannon diversity of the sequencing data, accounting for only 20% of the real diversity (shown in red). The best fit of the sequencing data with or without GCN using RSS compared with the mock composition is shown in bold

**Fig. 1:**
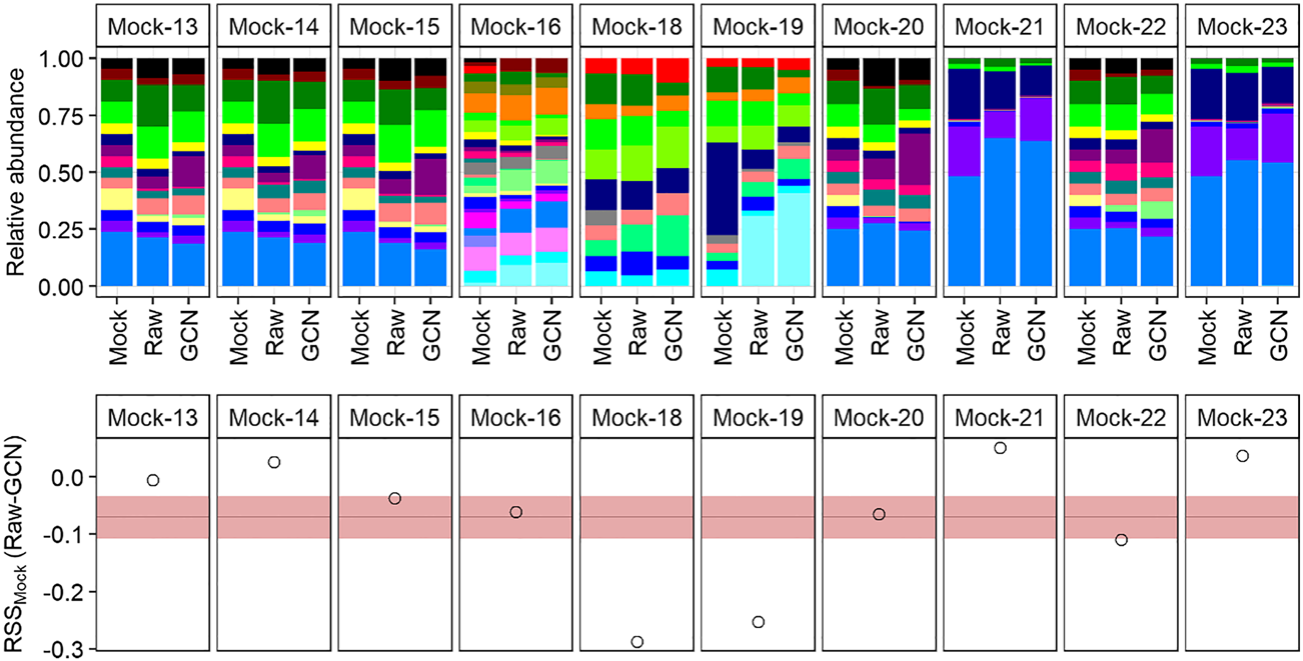
Microbial community structure as relative abundance of microbial genera (legend shown in Supplementary Fig. S1) and the difference between the residual sum squares (RSS) between 16S rRNA sequencing data without (raw) and with gene copy number normalization (GCN) compared with the mock community structure (Mock)

The average gene copy number in bacteria in the database using 152 bacterial genera was threefold higher with 5.29 (SE = 0.21) than the previously determined average for bacteria of 1.8. Using the higher average gene copy number would result in a similar representation of Mock-19 compared with the raw sequencing data (data not shown). We therefore suggest the reconsideration of 1.8 16S gene copies in bacteria as standard for unidentified sequences. Otherwise, GCN provided a picture closer to reality in the mock communities of lowest Shannon diversity (Mock-21 and Mock-23) with Mock-14 being the only exception with high diversity and a better fit with GCN. In Mock-14, all of the 18 genera from the mock community have a known gene copy number (available in the RDP GCN database), and both unidentified ASVs (0.04%) and ASVs assigned to other genera than present in the mock community (1.02%) make up a small proportion of the total ASV counts. The scenarios where GCN provides a better picture than the raw sequencing data therefore seemed artificial, given that the α-diversity in environmental samples is much higher than in our mock communities in both terrestrial [17–20] and aquatic ecosystems [21, 22], and it appears unreasonable to assume perfect sequencing data as found for Mock-14 (Fig. 2).

**Fig. 2:**
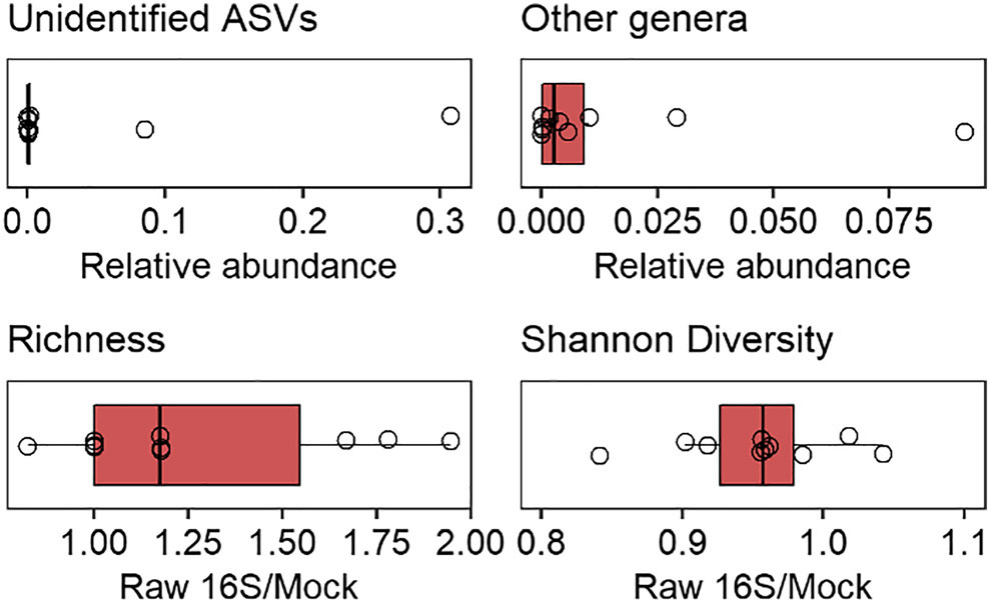
The difference between the richness and Shannon diversity of the 16S amplicon data without gene copy normalization and the mock community structure as well as the relative abundance of unidentified ASVs and ASVs assigned to other genera than present in the mock community as boxplots with median, lower and upper quartiles as well as minima and maxima

Correcting for 16S rRNA gene copy numbers in microbiome surveys still an unsolved problem [5]. The plasticity of the bacterial ribosome able to accommodate foreign 16S rRNA from diverse organisms as shown by horizontal gene transfer [23–27] also makes the use of GCN questionable. Our comparison of 16S amplicon data with the known structure of mock communities from purified DNA and plasmid vectors suggests that GCN only provided a better picture in artificial scenarios, e.g. low α-diversity or perfect sequencing data. However, different mock communities (from cells, RNA, proteins or metabolites), different primer pairs that target another region of the 16S rRNA gene and different processing pipeline may all yield different results. Importantly, predicting the as yet unknown 16S rRNA gene copy numbers [5, 28, 29] could be a viable approach to increase the fitness of GCN in amplicon sequencing. However, accounting for variance, e.g. by latent variable models, seems to be a more promising approach to understand the drivers of diversity [30] than correcting GCN sequence data. Noteworthy, we highlight the importance of quality checking publicly available mock community data to ensure high quality of future meta-analysis surveys.

## Supplementary Materials and Methods

### Data Generation

The community data was obtained from the *mockrobiata* database [7]. In total, 12 mock communities with known composition (as “taxonomy.csv” within “source” from *https://github.com/caporaso-lab/mockrobiota/tree/master/data*) containing both forward and reverse sequencing reads that target the 16S rRNA gene were obtained. The raw sequencing data was processed with *DADA2* v1.8 [8] using the *R* software to yield an ASV table that provides higher resolution than the traditional OTU table and records the number of times each exact ASV was observed in each sample (more information on the R script can be found in the Supplementary Material). The reads were truncated at position 230 for the forward and position 160 for the reverse read, respectively. The recovery of reads through the pipeline was tracked for each step in each sample (Supplementary Table S1). Mock-17 failed processing in the pipeline with our parameters and was removed from the analysis. For five communities (Mock-16, Mock-18, Mock-19, Mock-22 and Mock-23), many reads were lost after chimera removal (shown in red in Supplementary Table S1). In almost all cases, this is caused by primer sequences with ambiguous nucleotides that were not removed prior to data processing with *DADA2* [8]. Indeed, after removing the primers using the constant length of the forward primer 515f (*n* = 19) and the reverse primer 515f (*n* = 20) with the function *trimLeft = c(19,20)* within the function *filterAndTrim*, most of the sequences were retained after chimera removal, and the most abundant ASV length was 253. The taxonomy was assigned using the naïve Bayesian classifier method using the 16S database Silva (version 138 from May 6, 2020) and species level assignments based on exact matching between ASVs and sequenced reference strain [31, 32].

### Gene Copy Number Normalization and Statistical Analysis

The absolute ASV counts were divided by known 16S rRNA gene copy numbers from bacterial genomes obtained from the Ribosomal Database Project (RDP, Release 11, Update 5 from September 30, 2016) [10]. For bacterial genera without reported gene copy number, unidentified bacteria and other bacteria than present in the mock community, the average 16S rRNA gene copy number of 1.8 (*n* = 45, SE = 0.13) was used. For both with and without gene GCN, the absolute ASV counts were divided by the total number of recovered reads to obtain relative ASV abundances. The pipeline with the best fitting sequencing data was determined by residual sum squares (RSS) as deviation of the predicted abundance derived from the mock community composition from the empirical values of the 16S rRNA gene amplicon data from the difference of the *i*^*th*^ value between the mock community as *y*_*i*_ and the 16S rRNA gene sequencing without (raw) and with normalization (GCN) both as *f(x*_*i*_*)* given by Eq. 1. The relative amount of unidentified ASVs and ASVs assigned to genera that are not present in the mock community were estimated. Taxonomic richness was calculated as the number of different genera in each sample. Alpha diversity, as Shannon diversity, was calculated on the level of bacterial genera. Mock-12 was removed from the data analysis as the raw sequencing data was dominated by *Bacteroides* making up 90.7% of all reads while only present at 29.6% in the mock community and tremendously changing the evenness and thereby not only impacting the Shannon diversity of the community but also showing the highest RSS of all communities (shown in red in Table 1), which deemed the comparison with the other mock communities unreliable. Visualization was carried out in *R* using the package *ggplot2* [33].

1 $$ \mathrm{RSS}=\sum \limits_{i=1}^n{\left({y}_i-f\left({x}_i\right)\right)}^2 $$

## Electronic Supplementary Material

ESM 1 (PNG 918 kb)

High Resolution Image (TIF 426 kb)

ESM 2 (XLSX 9 kb)

ESM 3 (PDF 534 kb)
