# Supplementary figures and images for: 16S rRNA Gene Copy Number Normalization Does Not Provide More Reliable Conclusions in Metataxonomic Surveys
Source: Microb Ecol. 2020 Aug 29;81(2):535–9. doi: 10.1007/s00248-020-01586-7 (PMC7835310; doi:10.1007/s00248-020-01586-7)

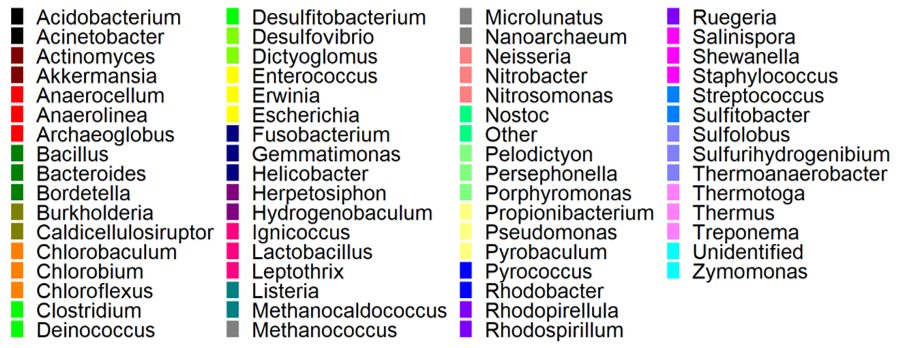

Supplement: Supplementary file 1 — (PNG 918 kb) [file 248_2020_1586_Fig3_ESM.png]

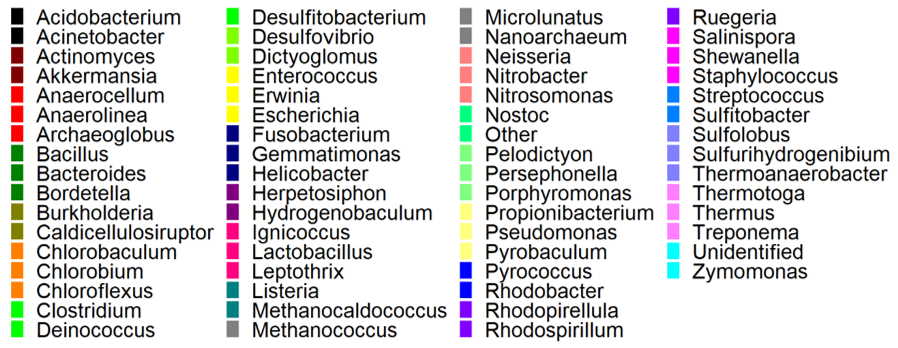

Supplement: Supplementary file 2 — High Resolution Image (TIF 426 kb) [file 248_2020_1586_MOESM1_ESM.tif]
