# Supplementary material for: 16S rRNA Gene Copy Number Normalization Does Not Provide More Reliable Conclusions in Metataxonomic Surveys
Source: Microb Ecol. 2020 Aug 29;81(2):535–9. doi: 10.1007/s00248-020-01586-7 (PMC7835310; doi:10.1007/s00248-020-01586-7)
Supplement: Supplementary file 4 — (PDF 534 kb) [file 248_2020_1586_MOESM3_ESM.pdf]

16S rRNA gene copy number normalization does not provide more reliable conclusions in metataxonomic surveys

Robert Starke, Victor Satler Pylro and Daniel Kumazawa Morais

8/14/2020

The following document provides the scripts for the analysis of the paper "16S rRNA gene copy number normalization does not provide more reliable conclusions in metataxonomic surveys" by Starke, Pylro and Morais.

At first, use "devtools" to download the recent version of "dada2". After setting the working directly with setwd and choosing the path where the files will be saved with path, the script is ready to run for the forward (saved as fnFs) and the reverse read (saved as fnRs) as example of mock community 13 from mockrobiota (https://github.com/caporaso-lab/mockrobiota). The reads should be cut at low quality (with truncLen) or when it contains primer sequences (with trimLfet).More information on DADA2 and troubleshooting can be obtained from https://benjjneb.github.io/dada2/tutorial.html.

```
library(dada2); packageVersion("dada2")

## Loading required package: Rcpp

## [1] '1.8.0'

path <- "C:/Users/starkr/Desktop/DADA2/"
fnFs <- sort(list.files(path, pattern="mock13-forward-read.fastq", full.names
= TRUE))
fnRs <- sort(list.files(path, pattern="mock13-reverse-read.fastq", full.names
= TRUE))
sample.names <- sapply(strsplit(basename(fnFs), "_"), `[`, 1)
plotQualityProfile(fnFs[1:2])

## Scale for 'y' is already present. Adding another scale for 'y', which
## will replace the existing scale.
```

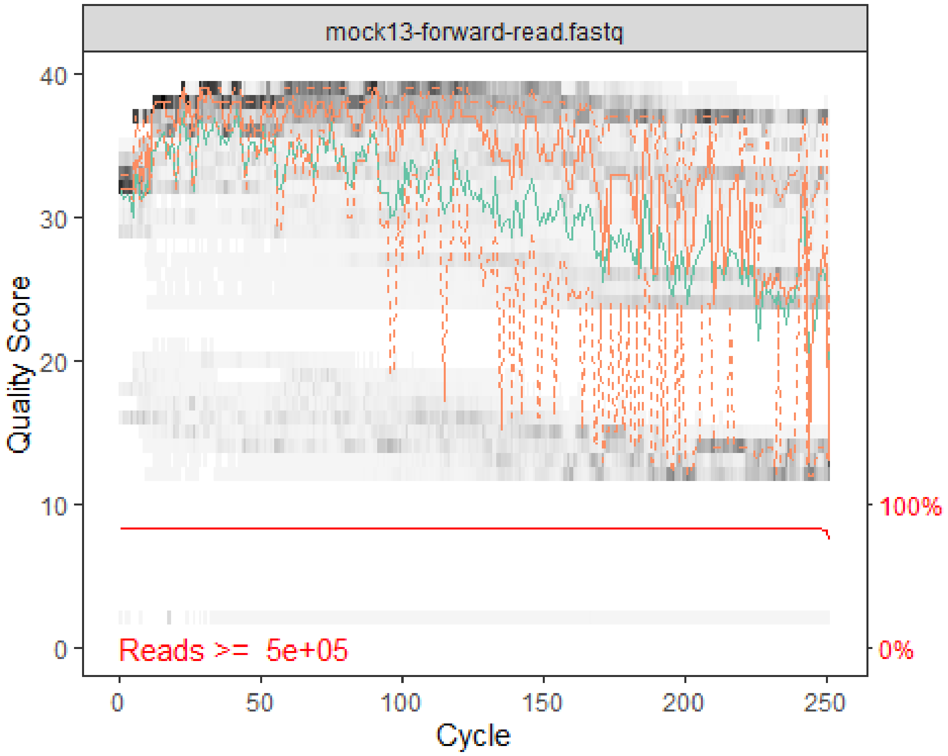


```
plotQualityProfile(fnRs[1:2])

## Scale for 'y' is already present. Adding another scale for 'y', which
## will replace the existing scale.
```

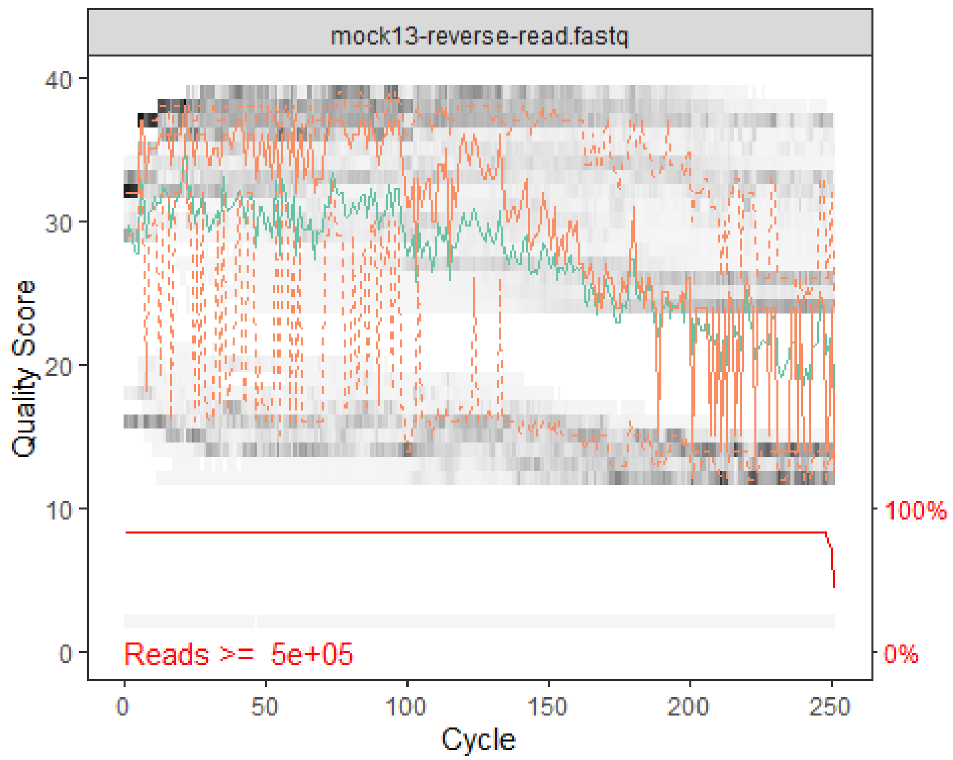


```
filtFs <- file.path(path, "filtered", paste0(sample.names, "_F_filt.fastq.gz"
))
filtRs <- file.path(path, "filtered", paste0(sample.names, "_R_filt.fastq.gz"
))
out <- filterAndTrim(fnFs, filtFs, fnRs, filtRs, truncLen=c(230,160),
                     maxN=0, maxEE=c(2,2), truncQ=2, rm.phix=TRUE,
                     compress=TRUE, multithread=F,trimLeft = c(19, 20)) # On
Windows set multithread=FALSE
head(out)

##                            reads.in reads.out
## mock13-forward-read.fastq    602819    300697

errF <- learnErrors(filtFs, multithread=F)

## 63447067 total bases in 300697 reads from 1 samples will be used for learn
ing the error rates.
## Initializing error rates to maximum possible estimate.
## selfConsist step 1 .
##    selfConsist step 2
##    selfConsist step 3
##    selfConsist step 4
##    selfConsist step 5
## Convergence after  5  rounds.

errR <- learnErrors(filtRs, multithread=F)
```

```
## 42097580 total bases in 300697 reads from 1 samples will be used for learn
ing the error rates.
## Initializing error rates to maximum possible estimate.
## selfConsist step 1 .
##    selfConsist step 2
##    selfConsist step 3
##    selfConsist step 4
##    selfConsist step 5
##    selfConsist step 6
## Convergence after  6  rounds.
```

```r
plotErrors(errF, nominalQ=TRUE)
```

```
## Warning: Transformation introduced infinite values in continuous y-axis

## Warning: Transformation introduced infinite values in continuous y-axis
```

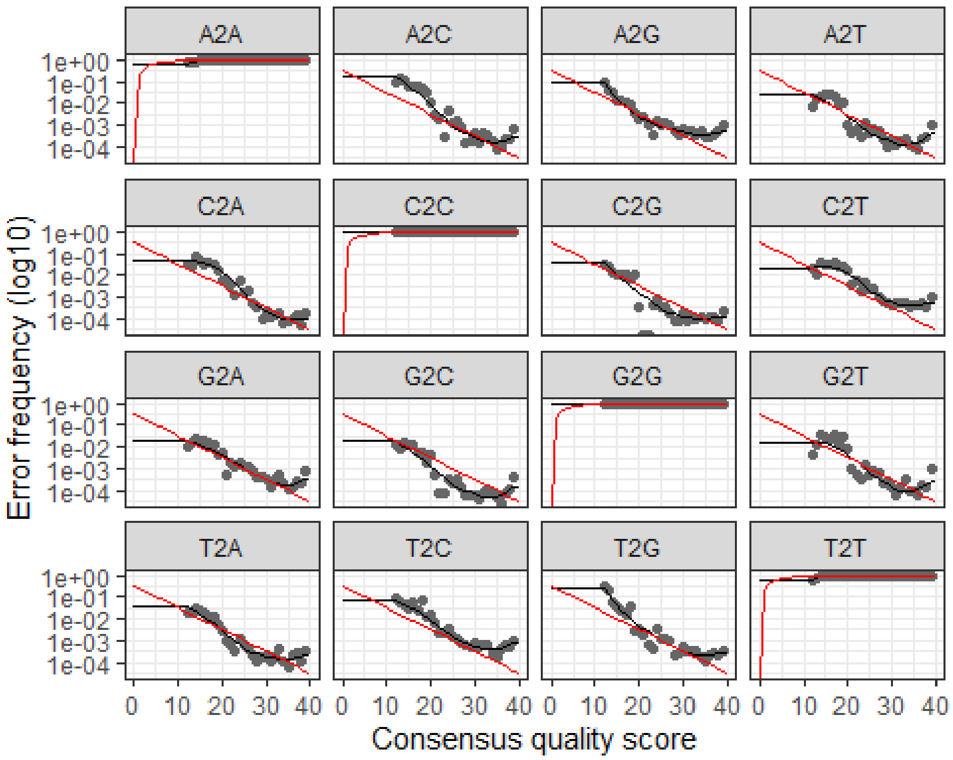


```r
derepFs <- derepFastq(filtFs, verbose=TRUE)
```

```
## Dereplicating sequence entries in Fastq file: C:/Users/starkr/Desktop/DADA
2//filtered/mock13-forward-read.fastq_F_filt.fastq.gz

## Encountered 62714 unique sequences from 300697 total sequences read.
```

```r
derepRs <- derepFastq(filtRs, verbose=TRUE)
```

```
## Dereplicating sequence entries in Fastq file: C:/Users/starkr/Desktop/DADA
2//filtered/mock13-forward-read.fastq_R_filt.fastq.gz
```

```
## Encountered 100007 unique sequences from 300697 total sequences read.

names(derepFs) <- sample.names

## Warning in `names<-`(`*tmp*`, value = "mock13-forward-read.fastq"): derep-
## class objects cannot be renamed.

names(derepRs) <- sample.names

## Warning in `names<-`(`*tmp*`, value = "mock13-forward-read.fastq"): derep-
## class objects cannot be renamed.

dadaFs <- dada(derepFs, err=errF, multithread=F)

## Sample 1 - 300697 reads in 62714 unique sequences.

dadaRs <- dada(derepRs, err=errR, multithread=F)

## Sample 1 - 300697 reads in 100007 unique sequences.

mergers <- mergePairs(dadaFs, derepFs, dadaRs, derepRs, verbose=TRUE)

## 275786 paired-reads (in 667 unique pairings) successfully merged out of 29
8879 (in 2379 pairings) input.

seqtab <- makeSequenceTable(mergers)

## The sequences being tabled vary in length.

dim(seqtab)

## [1]   1 667

table(nchar(getSequences(seqtab)))

##
## 213 214 215 332 333 335 336 337 338
##  11 132   1   3  49  15 152 297   7

seqtab.nochim <- removeBimeraDenovo(seqtab, method="consensus", multithread=F
, verbose=TRUE)

## Identified 586 bimeras out of 667 input sequences.

dim(seqtab.nochim)

## [1]  1 81

sum(seqtab.nochim)/sum(seqtab)

## [1] 0.963439

getN <- function(x) sum(getUniques(x))
track <- cbind(out, getN(dadaFs), getN(dadaRs), getN(mergers), rowSums(seqtab
.nochim))
```

```r
# If processing a single sample, remove the sapply calls: e.g. replace sapply
(dadaFs, getN) with getN(dadaFs)
colnames(track) <- c("input", "filtered", "denoisedF", "denoisedR", "merged",
"nonchim")
rownames(track) <- sample.names
head(track)
```

```
##                              input filtered denoisedF denoisedR merged
## mock13-forward-read.fastq 602819   300697    299536    299558 275786
##                           nonchim
## mock13-forward-read.fastq  265703
```

```r
taxa <- assignTaxonomy(seqtab.nochim, "C:/Users/starkr/Desktop/DADA2/MiSeq_SO
P/silva_nr_v138_train_set.fa", multithread=F)
taxa <- addSpecies(taxa, "C:/Users/starkr/Desktop/DADA2/MiSeq_SOP/silva_speci
es_assignment_v138.fa")
taxa.print <- taxa # Removing sequence rownames for display only
rownames(taxa.print) <- NULL
head(taxa.print)
```

```
##      Kingdom    Phylum             Class
## [1,] "Bacteria" "Firmicutes"       "Bacilli"
## [2,] "Bacteria" "Firmicutes"       "Bacilli"
## [3,] "Bacteria" "Proteobacteria"   "Gammaproteobacteria"
## [4,] "Bacteria" "Proteobacteria"   "Gammaproteobacteria"
## [5,] "Bacteria" "Campilobacterota" "Campylobacteria"
## [6,] "Bacteria" "Firmicutes"       "Clostridia"
##      Order                Family               Genus
## [1,] "Staphylococcales"   "Staphylococcaceae" "Staphylococcus"
## [2,] "Bacillales"         "Bacillaceae"       "Bacillus"
## [3,] "Pseudomonadales"    "Moraxellaceae"     "Acinetobacter"
## [4,] "Burkholderiales"    "Neisseriaceae"     "Neisseria"
## [5,] "Campylobacterales" "Helicobacteraceae" "Helicobacter"
## [6,] "Clostridiales"      "Clostridiaceae"    "Clostridium_sensu_stricto_1"
##      Species
## [1,] NA
## [2,] NA
## [3,] NA
## [4,] "meningitidis"
## [5,] "pylori"
## [6,] NA
```

This script was repeated for all samples. Then, the absolute ASV counts as result from the script were divided (or not) by the known 16S rRNA gene copy numbers from bacterial genomes obtained from the Ribosomal Database Project (RDP, Release 11, Update 5 from September 30, 2016). Taxonomic richness and Shannon diversity was calculated on the level of bacterial genera. Visualization was carried out using the package “ggplot2” (Wickham, 2017).
